# Fatty Acid Profiling in Facial Sebum and Erythrocytes From Adult Patients With Moderate Acne

**DOI:** 10.3389/fphys.2022.921866

**Published:** 2022-06-21

**Authors:** Ke Cao, Ye Liu, Ningning Liang, Xia Shen, Rui Li, Huiyong Yin, Leihong Xiang

**Affiliations:** ^1^ Department of Dermatology, Huashan Hospital, Fudan University, Shanghai, China; ^2^ CAS Key Laboratory of Nutrition, Metabolism and Food Safety, Shanghai Institute of Nutrition and Health (SINH), Chinese Academy of Sciences (CAS), Shanghai, China

**Keywords:** acne (acne vulgaris), sebum, fatty acid (composition), erythrocyte (human), insulin-like growth factor 1 (IGF1)

## Abstract

Fatty acid (FA) metabolism has been involved in acne vulgaris, a common inflammatory skin disease frequently observed in adolescents and adults, but it remains poorly defined whether the distributions or location of FA in facial sebum and those in the circulation differentially correlate with the disease. In a cohort of 47 moderate acne patients and 40 controls, sebum samples from forehead and chin areas were collected using Sebutape adhesive patches, and erythrocytes were separated from the fasting blood. Total FAs were analyzed by the gas chromatograph-mass spectrometry method. Compared to control female subjects, female patients showed increased levels of saturated fatty acids (SFAs) and monounsaturated fatty acids (MUFAs) from both facial areas, whereas decreased levels of polyunsaturated fatty acids (PUFAs) from chin areas were observed. Interestingly, the levels of docosahexaenoic acid (DHA) in the circulating erythrocytes were significantly decreased in male patients compared with control. In addition, DHA levels in erythrocytes were positively correlated with PUFAs from sebum only in male subjects. Furthermore, female patients with moderate acne had more severe sebum abnormity and chin-specific FA profiles, consistent with higher acne incidences than males in adulthood, especially in the chin areas. Importantly, serum insulin-like growth factor 1 (IGF-1) levels were positively correlated with SFAs and MUFAs from sebum only in male subjects. In summary, differential spatial FA distributions in facial sebum and correlation with those in erythrocytes and IGF1 levels in serum may shed some light on the pathology of acne in male and female adults.

## Introduction

Acne vulgaris is a multifactorial skin disease that frequently occurrs after puberty (Skroza et al., 2018). It continues to be a common and stressful skin problem even after teenage years and especially affects females at higher rates than males (Collier et al., 2008). Emerging evidence suggests that increased sebum production and alterations of sebum composition, including skin surface lipids (SSLs), are among the most pivotal factors in the pathogenesis of acne (Zouboulis et al., 2014; Camera et al., 2016; Zhou et al., 2018). SSL in sebum consists of diverse classes of lipids, among which fatty acids (FAs) are primarily responsible for the inflammatory and innate immune responses in the pathogenesis of acne (Ottaviani et al., 2010). Depending on the chemical structures, some FAs have pro-inflammatory and follicular keratinization properties (Katsuta et al., 2005; Choi et al., 2019), but other FAs show antibacterial and anti-inflammatory effects (Balic et al., 2020). A different ratio between saturated fatty acid (SFA) and monounsaturated fatty acid (MUFA) in the SSL of acne patients (Smith et al., 2008) was reported, suggesting that FA alterations in SSL could be considered causative factors of clinical signs of acne (Ottaviani et al., 2010).

Circulating polyunsaturated fatty acids (PUFAs) are usually detected in different lipoprotein particles, while the levels of erythrocyte PUFAs are known to reflect relatively long-term nutritional status (Arab, 2003) and are highly correlated with PUFA compositions in various tissues (Fenton et al., 2016). Furthermore, PUFA levels in the blood of acne patients are associated with a pro-inflammatory state, while supplementation with PUFA reverses acne lesions (Jung et al., 2014; Aslan et al., 2017). In addition, insulin-like growth factor 1 (IGF1) plays an important role in the pathogenesis of acne by inducing sebum overproduction (Ju et al., 2017). However, it remains to be poorly defined whether FA compositions in SSL in both genders and facial anatomical sites correlate with those in the circulation and IGF1 levels in serum.

In this study, we examined the sebum alteration of the forehead and chin in male and female patients with moderate acne, focusing on the correlations between spatial FA levels in facial sebum and those in blood circulation. Correlation of FA profiles with IGF1 levels was also made. Discovery of these clues on the FA compositions in acne may lay the ground for potential dietary interventions to alter the facial FA in the prevention and treatment of acne.

## Materials and Methods

### Study Population

Forty-seven patients with moderate acne and forty age- and gender-matched controls with normal facial skin were recruited between September and December 2020 in Huashan Hospital with the approval of the Huashan Hospital Ethics Committee, Fudan University, Shanghai, China. Written informed consent was obtained from all patients before the study. All participants were nonsmokers and did not consume alcohol. Patients with acne were selected from participants who had not previously received acne-related treatments for a minimum of 8 weeks prior to the enrollment. The clinical grading of patients and control subjects was assessed by dermatologists who followed the guidelines of acne vulgaris (Zaenglein et al., 2016). Patients of systemic diseases including polycystic ovarian syndrome (PCOS) and other skin disorders were excluded from this study. A survey of food frequency and 24-h dietary recall were conducted using a food frequency questionnaire (FFQ) (Roengritthidet et al., 2021).

### Sample Collection

Sebum was collected from the central forehead and chin using a Sebutape^®^ (CuDerm Corporation, Dallas, TX, United States). The Sebutape is an open-celled, microporous, hydrophobic, lipophilic adhesive strip that has been shown to be a reproducible technique for the collection of SSL (Nordstrom et al., 1986). The skin was degreased with a 75% alcohol swab and allowed to dry. A Sebutape that had been pre-weighed beforehand was placed onto the skin for 30 min. The tape was reweighed for the gravimetric assessment of sebum excretion rates (SERs, micrograms per square centimeter per minute). Table 1 summarizes the average SER for the study groups. The Sebutapes were stored at −80°C until further analysis. In line with the recommendations of the European Group for Efficacy Measurements on Cosmetics and Other Topical Products (Piérard et al., 2000), sebum collections were performed on all participants in the same examination room, where ambient temperature and humidity were kept constant. Sebutapes were applied between 11:00 am and 15:00 pm to avoid diurnal variations of sebum secretion. Peripheral blood was collected and centrifuged, and samples of both erythrocytes and plasma were stored at −80°C until analysis.

**TABLE 1:** Demographic details of control subjects and moderate acne patients.

Characteristic	Control subjects (*n* = 40)	Moderate acne patients (*n* = 47)
Male, N, (%)	18 (45)	17 (36)
Female, N, (%)	22 (55)	30 (64)
AGE, mean (SD), y	26.72 (3.154)	25.67 (3.273)^*NS*^
BMI, mean (SD), kg/m^2^	20.86 (1.578)	20.94 (1.570)^*NS*^
SER, mean (SD), μg/cm^2^/minute		
Total	6.166 (2.931)	6.577 (2.617)^*NS*^
Male	6.698 (3.085)	6.907 (2.971)^*NS*^
Female	5.711 (2.749)	6.349 (2.407)^*NS*^
Forehead	6.221 (2.522)	6.515 (2.666)^*NS*^
Chin	6.111 (3.323)	6.640 (2.597)^*NS*^
IGF1, mean (SD), μg/L		
Total	167.2 (33.96)	171.65 (33.19)^NS^
Male	155.5 (28.49)	167.14 (34.31)^NS^
Female	182.29 (34.5)	174.08 (32.31)^NS^

BMI, body mass index; SER, sebum excretion rate; IGF1, insulin-like growth factor 1.

### Sample Preparation

FAs were extracted according to a previously published protocol from our laboratory (Liu et al., 2021). In brief, a solvent mixture (chloroform/methanol, 2:1 v/v) was added with the addition of 0.025% antioxidant butylated hydroxytoluene and C21:0 (20 μg, internal standard). The extracts were vortexed for 5 min and then centrifuged at 2,000 g for 10 min. After discarding the Sebutapes, the extracts were dried under a gentle N_2_ flow and then derivatized to form fatty acid methyl esters (FAMEs) *via* the addition of 2 ml 2% H_2_SO_4_ in methanol and incubation at 80°C for 1 h. Next, 2 ml hexane and 500 μl ddH_2_O were added, and the upper phase was dried under the N_2_ flow. The samples were resuspended in 100 µl hexane prior to gas chromatography-mass spectrometry (GC-MS) analysis.

### GC-MS Analysis

A Shimadzu QP-2010 Ultra GC-MS was programed with an injection temperature of 250°C, injection split ratio of 1/20, and injection volume of 1 μl sample. The GC column was a 30 m × 0.25 mm × 0.25 mm HP-5ms. The amount of FA was quantified by using a standard response curve with the internal standard. The samples were analyzed in a random sequence. Quality control samples and blank control samples were injected for every 15 samples to ensure repeatability.

### Statistical Analysis

Statistical analysis was conducted using GraphPad Prism (Graph-Pad Software) and SPSS, version 26 (IBM Corporation). Continuous variables were expressed as means and standard deviations (SDs) and categorical data as percentage distributions. Student’s t test and analysis of variance test were used for quantitative data, and Fisher’s test was used to compare categorical data. A statistical probability of *p* < 0.05 was considered significant. Z-score was calculated and presented in a heatmap using the R package ComplexHeatmap v.2.8.0. Red in the heatmap represents that Z-score is more than 0, whereas blue represents that Z-score is less than 0. Spearman’s correlation coefficients between sebum within samples in each group were calculated and visualized by R package corrplot 0.9.0. Negative correlations were shown in blue, whereas positive correlations were in red. All the statistical analyses were performed using R v.4.1.0.

## Results

### Subject Demographics

Participants in the current study were 18–35 years old. Demographics of controls and subjects with acne are described in Table 1. Two groups were similarly distributed in terms of gender and age. The body mass index (BMI) in the two groups was within the normal range. To evaluate sebometry, the parameter associated with acne pathogenesis including the SER was determined for each participant. The SER of acne patients was all increased than that of control subjects regardless of the genders and facial sites but did not reach statistical significance. To minimize the effects of SER values on sebum chemometrics, the levels of FA in all samples were normalized to the weight of sebum.

### FA Profiles in Sebum

**FIGURE 1:**
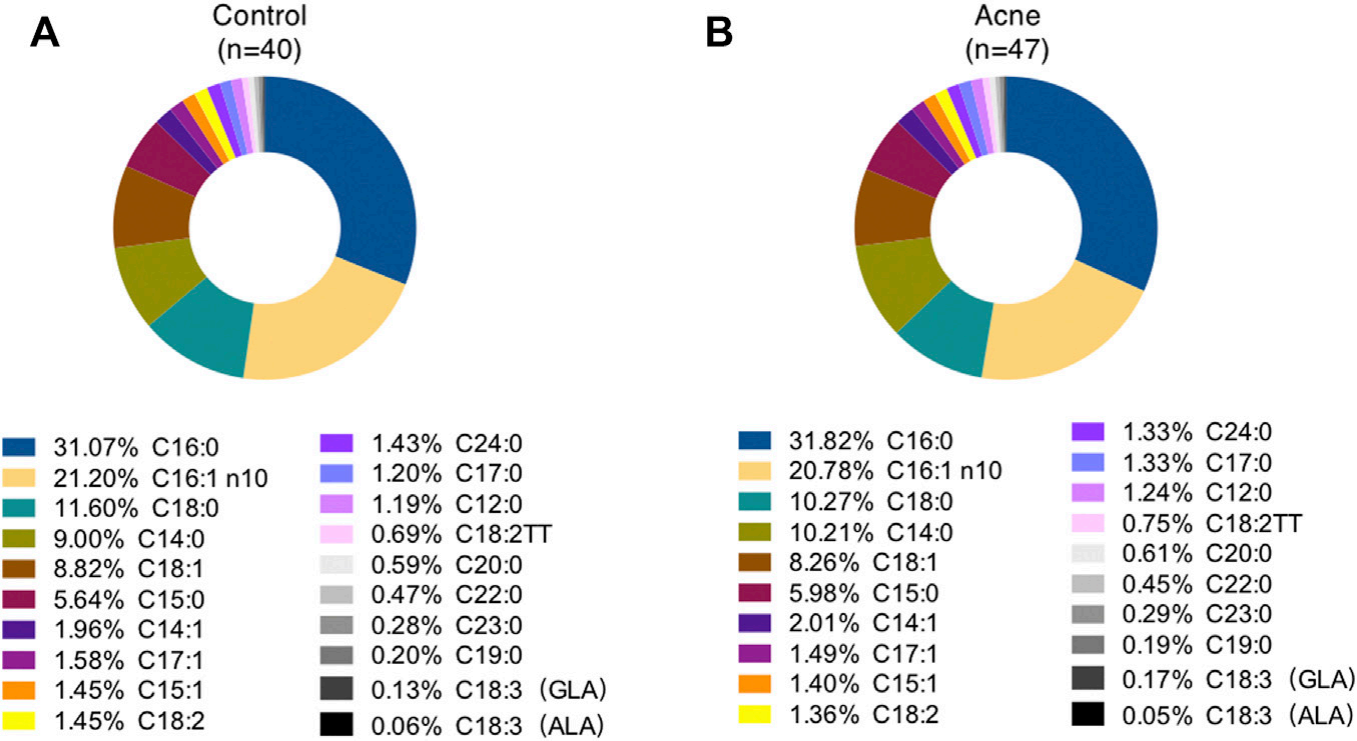
Percentage of total fatty acids in sebum from control subjects **(A)** and acne patients **(B)**. GLA, γ-linolenic acid; ALA, α-linolenic acid.

**FIGURE 2:**
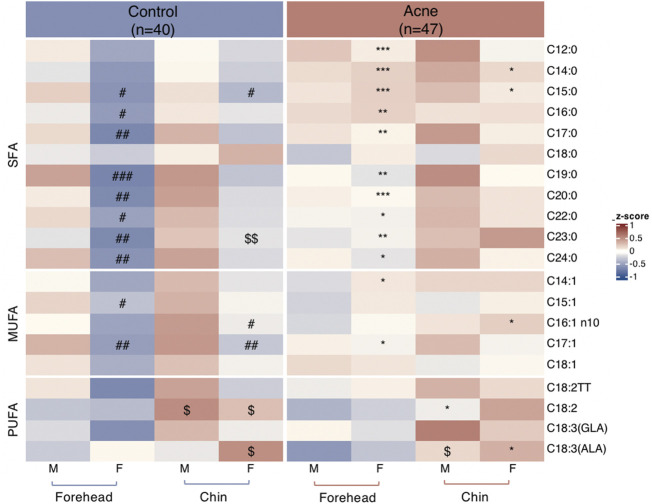
Heatmaps of quantified fatty acids levels normalized by Z-score from sebum on forehead and chin between males (M) and females (F) in control and acne groups. *p < 0.05, **p < 0.01, and ***p < 0.001 vs. the gender- and site-matched control group; ^#^ *p* < 0.05, ^##^ *p* < 0.01, and ^###^ *p* < 0.001 vs. site-matched male control group; ^$^ *p* < 0.05 and ^$$^ *p* < 0.01 vs. gender- and group-matched subjects.

A total of twenty FAs in SSL were quantified in the GC-MS analysis (Supplementary Table S1). Palmitic acid (PA, C16:0), sapienic acid (SA, C16:1n-10), stearic acid (C18:0), myristic acid (C14:0), and oleic acid (OA, C18:1) are the top five most abundant FAs in the sebum, accounting for about 31%, 21%, 11%,10%, and 8%, respectively, for control subjects (Figure 1A) and acne patients (Figure 1B), consistent with previous reports (Akaza et al., 2014). However, none of these FAs between the two groups was statistically significant. Moreover, 11 SFA, five MUFA, and four PUFA were detected in our method. Heatmaps of the average amounts of sebum lipids in the forehead and chin of both genders between the control and acne groups are reported in Figure 2. First, comparisons within each group were made. In the control group, SFA (C15:0, C16:0, C17:0, C19:0, C20:0, C22:0, C23:0, C24:0) and MUFA (C15:1, C17:1) from forehead were decreased in female subjects compared to male ones (^#^ *p* < 0.05). Meanwhile, the levels of SFA (C15:0) and MUFA (C16:1n10, C17:1) from the chin were also lower in female subjects than in males (^#^ *p* < 0.05). However, these differences in SFA and MUFA between the two genders did not differ within the acne group. Moreover, in the control group, the level of linoleic acid (C18:2, LA) from the chin of both genders was higher than that of the forehead (^$^ *p* < 0.05). C18:3 (α-linolenic acid, ALA) was also increased in the chin of women (^$^ *p* < 0.05). However, within the acne group, when gender and site were taken into consideration, only men had higher levels of ALA (^$^ *p* < 0.05) in the chin than the forehead. Next, comparisons were made between the two groups. Female acne patients had more FA alterations in the sebum (shown as ^*^ *p* < 0.05). In detail, in the forehead of female subjects, all detected SFA (except C18:0) and two MUFA (C14:1 and C17:1) were significantly increased compared to those of control females (*p < 0.05). Meanwhile, markedly increased levels of SFA (C14:0, C15:0) and MUFA (C16:1n10) and decreased level of PUFA (ALA) were detected in the chin of female acne patients than in control female subjects (*p < 0.05). In addition, male patients had lower levels of PUFA (C18:2) in the chin in the acne group than in the control group (*p < 0.05). Overall, the FA abnormality of facial sebum in acne patients was mainly found in females in a spatial and gender-specific manner.

### FA Profile of Erythrocytes in Subjects

We separated blood samples into erythrocytes and plasma fractions. The levels of erythrocytes FA are known to reflect relatively long-term nutritional status (Arab, 2003). Thirteen FAs were detected in erythrocytes, including six SFAs, two MUFAs, and five PUFAs (Table 2). We found that levels of docosahexaenoic acid (C22:6, DHA) in erythrocytes were significantly decreased in male patients with moderate acne than in control male subjects (*p < 0.05). Interestingly, female patients with acne showed higher levels of DHA than male patients (^#^ *p* < 0.05). However, none of the FA in plasma had significant differences between control and acne groups in both genders (Supplementary Table S2).

**TABLE 2:** Fatty acid profile of erythrocytes between control and acne groups.

FA of erythrocyte (μg/μl, mean ± SD)				
	Control subjects (*n* = 40)		Moderate acne patients (*n* = 47)	
	Male	Female	Male	Female
C12:0	0.03 ± 0.01	0.05 ± 0.03	0.05 ± 0.03	0.12 ± 0.07
C14:0	0.47 ± 0.12	0.51 ± 0.17	0.50 ± 0.23	0.78 ± 0.37
C15:0	0.24 ± 0.06	0.25 ± 0.08	0.21 ± 0.04	0.37 ± 0.20
C16:0	48.61 ± 6.58	46.68 ± 6.86	46.17 ± 6.04	50.50 ± 6.12
C17:0	0.47 ± 0.05	0.49 ± 0.07	0.38 ± 0.10	0.51 ± 0.07
C18:0	14.63 ± 1.32	14.35 ± 2.58	14.48 ± 2.85	16.33 ± 3.67
C16:1	0.36 ± 0.18	0.37 ± 0.12	0.34 ± 0.29	0.53 ± 0.35
C18:1	33.00 ± 4.74	29.41 ± 5.72	32.28 ± 5.34	32.55 ± 4.35
C18:2	26.44 ± 4.03	24.91 ± 6.34	26.17 ± 2.71	28.22 ± 5.91
C18:3 (ALA)	0.27 ± 0.07	0.22 ± 0.09	0.27 ± 0.07	0.33 ± 0.15
C20:4	9.80 ± 1.27	10.10 ± 1.82	9.45 ± 1.63	10.14 ± 2.18
C20:5	0.57 ± 0.15	0.52 ± 0.15	0.54 ± 0.17	0.77 ± 0.27
C22:6	5.48 ± 0.55	6.25 ± 1.17	4.58 ± 0.51*	7.01 ± 1.26^#^

ALA, α-linolenic acid; *p < 0.05 vs. the gender-matched control subjects, ^#^ *p* < 0.05 vs. the group-matched male subjects.

### Correlation of FA Levels With Those in the Sebum and Blood

**FIGURE 3:**
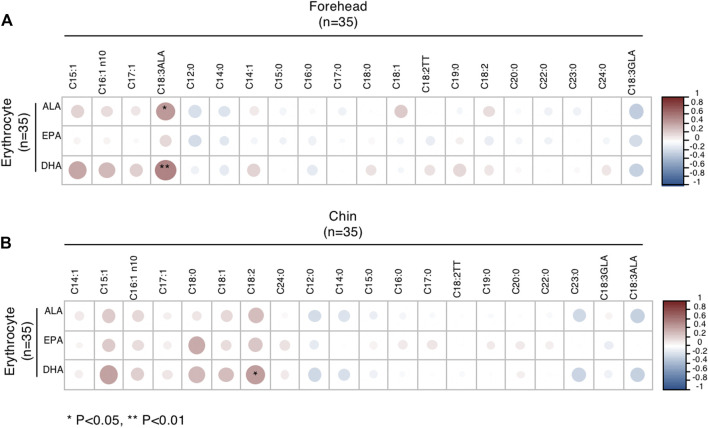
Correlations between Omega-3 PUFA of erythrocytes and FA of sebum from the forehead **(A)** and chin **(B)** in male subjects. *p < 0.05 and **p < 0.01.

As we observed that DHA in erythrocytes had specific differences in males, we further analyzed the correlation of Omega-3 PUFAs in erythrocytes and sebum. In male subjects, ALA and DHA levels of erythrocytes were positively correlated with ALA levels in sebum from the forehead (Figure 3A). Meanwhile, DHA levels of erythrocytes positively correlated with LA levels from the chin (Figure 3B). However, the patterns of correlation were different in females (Supplementary Figure S1).

### Correlation of IGF1 Levels and FA Composition From Sebum

**FIGURE 4:**
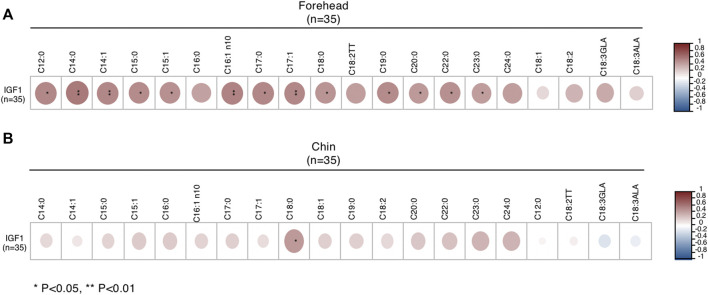
Correlations between IGF1 levels and FA of sebum from forehead **(A)** and chin **(B)** in male subjects. *p < 0.05 and **p < 0.01.

Next, we examined the correlation between IGF1 levels with sebum FAs and found that IGF1 levels were increased in male patients with moderate acne, but it did not reach statistical significance (Table 1). We further analyzed the correlation of IGF1 levels and FA composition from sebum. In male subjects, IGF1 levels were positively correlated with SFA (C12:0, C14:0, C15:0, C17:0, C18:0, C19:0, C20:0, C22:0, C23:0) and MUFA (C14:1, C15:1, C16:1n10, C17:1) levels in sebum from the forehead (Figure 4A). Meanwhile, IGF1 levels were positively correlated with SFA (C18:0) levels from the chin (Figure 4B). However, IGF1 levels showed no correlation with FA composition from sebum in females (Supplementary Figure S2). Previous studies showed that milk consumption was positively associated with circulating IGF-1 levels (Melnik and Schmitz, 2009). We found that the frequent consumption of milk, 250 ml or more per day, was associated with acne in categorical analysis in both genders, although it did not reach statistical significance (Table 3).

**TABLE 3:** Categorical analysis of milk consumption and acne.

Characteristic	Control subjects (*n* = 40)	Moderate acne patients (*n* = 47)	Odds ratio (95% CI)	*p*-value
Any kind of milk, n (%)				
Total				
<250 ml/day	31 (77.5)	29 (61.7)	2.138 (0.793–5.237)	0.163
≥250 ml/day	9 (22.5)	18 (38.3)		
Male				
<250 ml/day	15 (83.3)	9 (52.9)	4.444 (0.959–17.86)	0.075
≥250 ml/day	3 (16.7)	8 (47.1)		
Female				
<250 ml/day	16 (72.7)	20 (66.7)	1.333 (0.417–4.429)	0.765
≥250 ml/day	6 (27.3)	10 (33.3)		

## Discussion

Acne is a common dermatosis characterized by increased sebum production and inflammatory response (Melnik, 2015). It frequently occurs in teenagers and continues to late adolescence or early adulthood (Collier et al., 2008). Teenagers are prone to have numerous inflammatory and non-inflammatory comedonal lesions in T-zone (forehead, nose, and upper cheeks); however, the female type of adult acne presents deep-seated, long-lasting small nodules and cysts in the U-zone (chin, jawline, and neck) (Holzmann and Shakery, 2014; Dreno et al., 2018). Emerging studies have focused an SSL abnormity in both juvenile acne and adult acne (Camera et al., 2016; Zhou et al., 2018) and found that FA variations in sebum play a vital role in the induction of acne inflammation (Zouboulis et al., 2014). Furthermore, FAs in facial sebum can be affected by many factors, including acne severity, age, gender, anatomical site, circadian rhythm, and drug application (Ní Raghallaigh et al., 2012; Cui et al., 2018; Jia et al., 2019; Zhou et al., 2020a; Zhou et al., 2020b; Zhou et al., 2020c). However, the FA alterations in facial sebum remain to be clearly studied. In this study, we systematically analyzed FA levels in facial sebum in the forehead and chin area and in erythrocytes from both male and female acne patients, compared with their gender- and aged-matched control subjects. To the best of our knowledge, this study represents the first study on Chinese populations with moderate acne, who have distinct lifestyles, including dietary habits and environmental exposure, from the Western countries.

FAs can have both pro-inflammatory and anti-inflammatory effects in the context of acne pathogenesis (Balic et al., 2020). SFAs, such as palmitic acid (PA, C16:0), can induce the production of proinflammatory cytokines in sebocytes, keratinocytes, and macrophages (Zhou et al., 2013; Choi et al., 2019). On the other hand, unsaturated fatty acids (UFAs), especially MUFA, including sapienic acid (SA, C16:1n-10), palmitoleic acid (POA, C16:1), and oleic acid (OA, C18:1), have bactericidal activity (Wille and Kydonieus, 2003). However, the potential role of SA which is unique to human sebum in the etiology of acne is still controversial and remains to be elucidated. SA can replace LA and result in comedogenesis besides its antimicrobial function (Stewart et al., 1986; Perisho et al., 1988). Furthermore, POA and OA induce calcium influx into keratinocytes and cause abnormal differentiation of the epidermis that characterizes acne (Katsuta et al., 2005). In the current study, we substantiated that levels of SFA and MUFA from sebum were at higher levels in adult female acne patients than in control females, whereas no significant difference in these FAs was observed in male subjects. These observations might partly explain the increased rates of acne in women than men after puberty (Collier et al., 2008). Furthermore, we observed markedly increased levels of SA in the chin of female patients, consistent with the fact that adult women have a specific appearance of U-zone acne.

Notably, FAs consisted of odd carbon that may come from dairy and intestinal bacteria. For example, pentadecanoic acid (C15:0) and heptadecanoic acid (C17:0), which account for only a small proportion of total saturated fatty acids in milk fat and ruminant meat, can also be synthesized endogenously from gut-derived propionic acid (C3:0) (Pfeuffer and Jaudszus, 2016). Fatty acid desaturase 2 (FADS2), highly expressed in the human sebaceous gland, has been shown to convert C17:0 to C17:1 (Wang et al. 2020).

PUFAs are classified as Omega-3 and Omega-6, such as ALA and LA, respectively (Zouboulis et al., 2014). The therapeutic effect of ALA has been attributed to its anti-inflammatory activity (Balic et al., 2020). LA can attenuate the development of comedonal acne, and topical application of LA reduces microcomedones (Nicolaides et al., 1972; Letawe et al., 1998). Interestingly, we found that in the control group, female subjects have much more ALA and LA in the chin than the forehead, but this trend disappeared in the acne group. Instead, decreased levels of ALA in the chin of females acne patients compared to control females were observed. These data may partially explain why acne lesions are likely to spread on the chin of adult women (Dreno et al., 2018; Muguet Guenot et al., 2018). Thus, it is tempting to speculate that topical application of ALA or LA may be beneficial specifically in female acne patients.

Erythrocytes FAs are known to reflect relatively long-term dietary status (Arab, 2003) and are highly correlated with FA compositions in various tissues (Fenton et al., 2016). Hence, we also analyzed FA profiles of erythrocytes and plasma in acne patients and control subjects. Our data showed the decreased levels of Omega-3 FA in male patients, especially DHA in erythrocytes, compared to the control. It has been reported that an aggravation of acne and changes of FA in sebum were associated with increased intake of a Western diet that contains more saturated fat (Macdonald, 1964; Burris et al., 2014). The ratio of Omega-6/Omega-3 is 15:1 to 16:1 in a Western diet, while the recommended ratio differs from 1:1 up to 4:1 Balic et al., (2020). A high intake of these “danger signals” can activate the nutrient-sensitive kinase mechanistic target of rapamycin complex 1 (mTORC1), which stimulates sterol response element binding protein-1 (SREBP-1) and then upregulates stearoyl-CoA-desaturase (SCD), enhancing the proportion of MUFA in sebum triglycerides during the pathogenesis of acne (Melnik, 2015). Smith RN et al. (Smith et al., 2008) reported that a low glycemic load diet on acne patients was beneficial because it showed a decreased desaturase activity, contributing to a low level of MUFA in SSL. Furthermore, decreased PUFA (eicosapentaenoic acid, EPA) level in acne reveals the presence of a proinflammatory state (Aslan et al., 2017), while supplementing with EPA may be used as adjuvant treatments for acne patients (Jung et al., 2014). In our study, we tried to correlate FA profiles of erythrocytes and sebum and found a distinct pattern of correlation between females and males, suggesting that dietary intervention can be a viable therapeutic approach for male acne patients.

The relationship between IGF1 and acne remains controversial. Some studies reported that IGF1 levels were increased in acne patients (Agamia et al., 2016), while others showed no significant difference in IGF1 levels between acne patients and controls (Cappel et al., 2005; Aktas Karabay et al., 2020). Moreover, Vora et al. (2008) found that serum IGF1 levels were positively correlated with facial sebum excretion and acne lesion counts. However, the correlation between IGF1 levels and FA compositions of facial sebum has never been reported. In this study, we demonstrated that IGF1 levels were positively correlated with SFA and MUFA levels in sebum from male patients with moderate acne. Interestingly, milk consumption appears to be associated with increased IGF1 levels in serum, consistent with previous reports in which IGF1 was positively associated with acne severity in adults (Norat et al., 2007; Roengritthidet et al., 2021) Our study suggests that diets with high IGF1 should be seriously considered during the outbreak of acne due to their direct effect on FA composition of facial sebum, especially for male acne patients.

There are some limitations in this study. The study population was limited to moderate acne patients after puberty and did not include teenagers and mild and severe acne patients. Because the BMI of the enrolled participants was normal and toward lean bodies, observations from this study may not reflect overweight and obese populations. Due to the low levels of EPA and DHA in sebum, these two essential FAs were below the detection limit in sebum FA profiling. Furthermore, inflammatory parameters were not considered in the study. Milk consumption was not categorized into specific milk types, and other dietary intakes need to be included in the dietary questionnaire. Last, this cross-sectional study does not provide dynamic changes during the pathogenesis of acne, and future longitudinal study with multiple time points for follow-ups may provide more mechanistic insights.

In summary, our study has provided evidence that FA alterations in facial sebum in female acne patients can be partially responsible for their higher incidence than males in adulthood. Moreover, altered FA compositions of different anatomical sites resulted in an increased inflammation environment characteristic of U-zone acne in adult women. In addition, we observed distinct patterns of correlations between Omega-3 PUFA in erythrocytes and sebum and correlations between IGF1 levels in serum and sebum in male acne patients. Overall, our study suggests that gender- and site-specific topical application of FA can be used as sebum-modifying treatments, especially for Omega-3 PUFA.

## Data Availability

The original contributions presented in the study are included in the article/Supplementary Material; further inquiries can be directed to the corresponding authors.
