# Supplementary material for: Fatty Acid Profiling in Facial Sebum and Erythrocytes From Adult Patients With Moderate Acne
Source: Front Physiol. 2022 Jun 21;13:921866. doi: 10.3389/fphys.2022.921866 (PMC9253609; doi:10.3389/fphys.2022.921866)
Supplement: Supplementary file 1 [file DataSheet1.docx]

Supplementary Material

**Supplementary Table 1. Levels of fatty acids in sebum on forehead and chin of study subjects**

|  | **Control (μg /mg, mean±SD)**  **(n=40)** | | | | **Acne (μg /mg, mean±SD)**  **(n=47)** | | | |
| --- | --- | --- | --- | --- | --- | --- | --- | --- |
|  | Forehead | | Chin | | Forehead | | Chin | |
|  | Male | Female | Male | Female | Male | Female | Male | Female |
| C12:0 | 2.46±1.85 | 1.62±0.75 | 2.33±1.89 | 2.04±1.45 | 2.72±1.54 | 2.45±1.09 | 3.13±2.82 | 2.29±1.05 |
| C14:0 | 16.52±7.09 | 12.38±5.44 | 17.88±8.26 | 15.35±10.39 | 19.06±10.46 | 19.55±7.77 | 21.34±16.42 | 19.2±8.85 |
| C15:0 | 11.62±5.87 | 7.38±2.77 | 11.2±4.34 | 8.57±5.37 | 11.11±5.94 | 11.62±4.8 | 12.11±8.65 | 10.95±5.2 |
| C16:0 | 54.54±21.52 | 41.33±13.67 | 59.84±25.6 | 53.71±34.01 | 61.61±41.36 | 62.82±28.26 | 60.53±38.82 | 60.96±30.77 |
| C17:0 | 2.4±1.08 | 1.45±0.54 | 2.62±1.04 | 1.86±1.23 | 2.35±1.22 | 2.26±1.04 | 2.76±1.78 | 2.29±1.2 |
| C18:0 | 17.88±8.89 | 16.25±5.9 | 19.46±10.39 | 22.39±17.37 | 15.86±7.64 | 19.49±9.24 | 17.02±7.83 | 20.46±11.99 |
| C19:0 | 0.46±0.22 | 0.23±0.11 | 0.47±0.22 | 0.31±0.23 | 0.38±0.22 | 0.34±0.18 | 0.48±0.29 | 0.37±0.2 |
| C20:0 | 1.13±0.62 | 0.7±0.23 | 1.34±0.71 | 0.98±0.64 | 1.12±0.67 | 1.08±0.44 | 1.26±0.81 | 1.15±0.62 |
| C22:0 | 0.93±0.6 | 0.56±0.23 | 1.01±0.52 | 0.75±0.49 | 0.82±0.76 | 0.81±0.39 | 1.02±0.89 | 0.9±0.59 |
| C23:0 | 0.55±0.37 | 0.27±0.13 | 0.74±0.44 | 0.55±0.37 | 0.7±0.51 | 0.6±0.52 | 0.76±0.78 | 0.86±0.85 |
| C24:0 | 3.01±1.76 | 1.68±0.73 | 3.22±1.54 | 2.3±1.7 | 2.64±1.66 | 2.39±1.1 | 3.12±2.18 | 2.64±1.55 |
| C14:1 | 3.66±1.77 | 2.74±1.65 | 4.31±2.59 | 3.44±2.48 | 3.34±1.61 | 3.88±1.59 | 4.04±2.79 | 4.04±2.03 |
| C15:1 | 2.99±1.66 | 2.07±1.48 | 3.31±2.33 | 2.58±2.62 | 2.24±1.35 | 2.81±1.86 | 2.45±1.62 | 2.76±2.11 |
| C16:1 n10 | 39.84±18.93 | 28.3±18.27 | 50.49±28.29 | 37.88±29.09 | 35.29±20.12 | 39.47±18.78 | 42.29±28.58 | 44.62±25.84 |
| C17:1 | 3.38±1.73 | 1.99±1.05 | 3.6±1.72 | 2.38±1.7 | 2.91±1.62 | 2.77±1.28 | 3.32±2.5 | 2.77±1.43 |
| C18:1 | 18.18±13.72 | 11±6.41 | 20.31±13.03 | 15.97±13.51 | 18.78±23.64 | 18.06±15.19 | 15.48±11.05 | 16.47±10.75 |
| C18:2TT | 1.45±0.7 | 0.86±0.45 | 1.69±0.84 | 1.19±0.8 | 1.31±0.64 | 1.4±0.58 | 1.63±1.29 | 1.48±0.81 |
| C18:2 | 1.63±0.94 | 1.51±0.85 | 3.55±2.26 | 2.98±1.79 | 1.4±0.67 | 1.76±1.03 | 2.16±1.48 | 3.29±4.68 |
| GLA | 0.35±0.24 | 0.28±0.27 | 0.46±0.21 | 0.33±0.24 | 0.36±0.23 | 0.31±0.14 | 0.48±0.47 | 0.39±0.22 |
| ALA | 0.13±0.08 | 0.19±0.13 | 0.23±0.3 | 0.38±0.24 | 0.08±0.05 | 0.13±0.12 | 0.29±0.26 | 0.29±0.34 |

**Supplementary Table 2. The fatty acid profiles in plasma of study subjects**

| **FA of plasma (μg/μl, mean±SD)** | | | |  |
| --- | --- | --- | --- | --- |
|  | Control Subjects  (n=40) | | Moderate Acne Patients  (n=47) | |
|  | Male | Female | Male | Female |
| C12:0 | 0.08±0.03 | 0.13±0.08 | 0.10±0.08 | 0.21±0.20 |
| C14:0 | 1.15±0.36 | 1.21±0.41 | 1.21±0.96 | 1.16±0.42 |
| C15:0 | 0.41±0.15 | 0.46±0.30 | 0.29±0.14 | 0.35±0.13 |
| C16:0 | 48.52±9.40 | 42.57±4.96 | 44.47±7.24 | 44.95±5.18 |
| C17:0 | 0.40±0.11 | 0.41±0.07 | 0.33±0.08 | 0.42±0.12 |
| C18:0 | 14.93±2.40 | 13.31±1.48 | 13.07±1.55 | 14.65±2.05 |
| C16:1 | 3.44±0.85 | 3.41±0.84 | 3.56±1.22 | 2.97±0.85 |
| C18:1 | 44.30±10.10 | 38.12±9.31 | 46.40±10.46 | 38.49±6.54 |
| C18:2 | 68.47±10.96 | 62.31±10.19 | 63.58±7.74 | 65.99±9.88 |
| C18:3(ALA) | 1.34±0.43 | 1.28±0.58 | 1.12±0.38 | 1.67±0.54 |
| C20:4 | 7.21±2.01 | 6.79±2.36 | 6.44±1.31 | 6.57±1.50 |
| C20:5 | 0.62±0.29 | 0.50±0.19 | 0.40±0.16 | 0.67±0.31 |
| C22:6 | 3.50±1.27 | 3.43±0.64 | 2.45±0.31 | 3.90±1.15 |


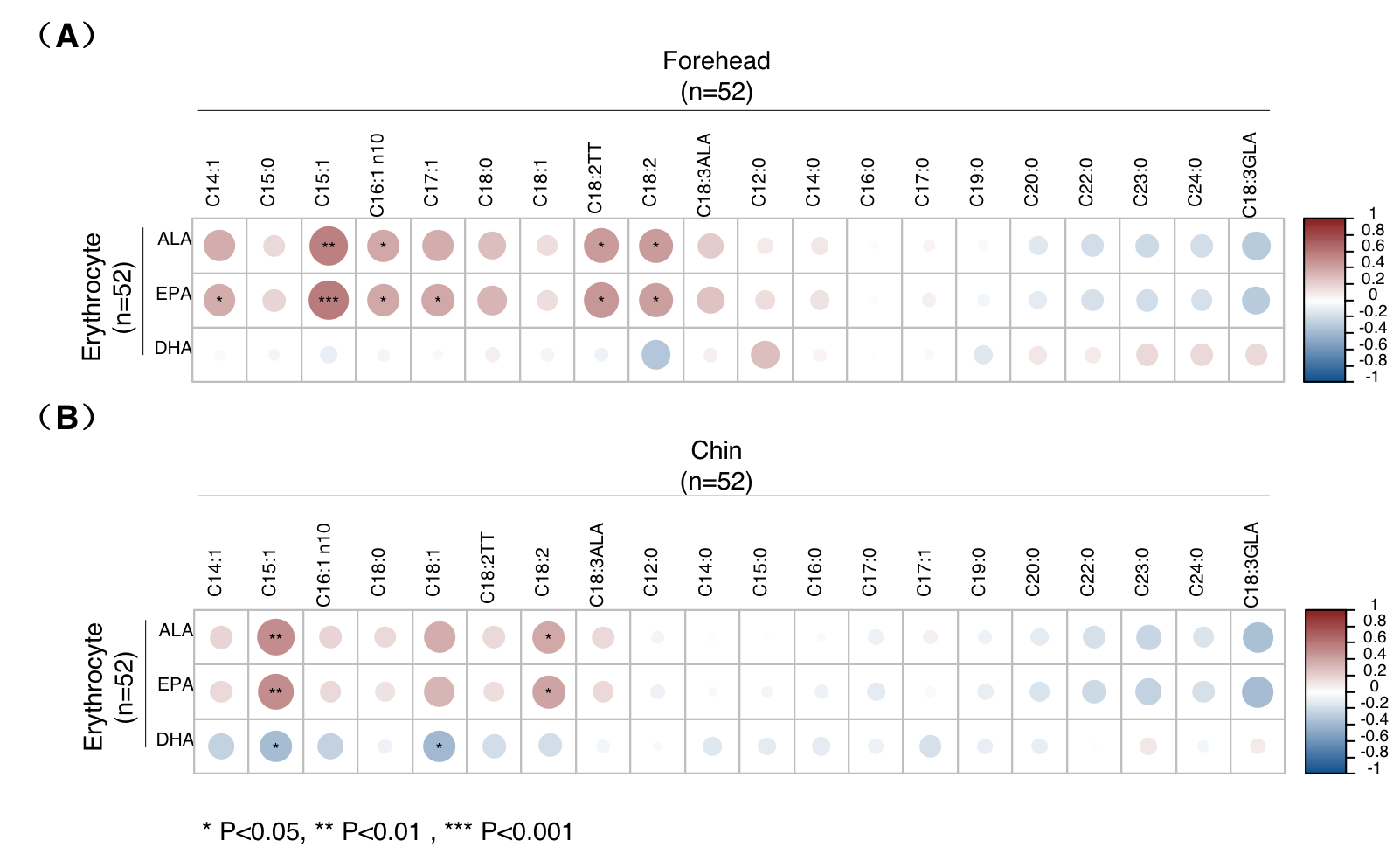


**Supplementary Figure 1.** **Correlations between Omega-3 PUFA of erythrocyte and FA of sebum from forehead (A) and chin (B) in female subjects.** *P< 0.05, **P < 0.01, ***P < 0.001.


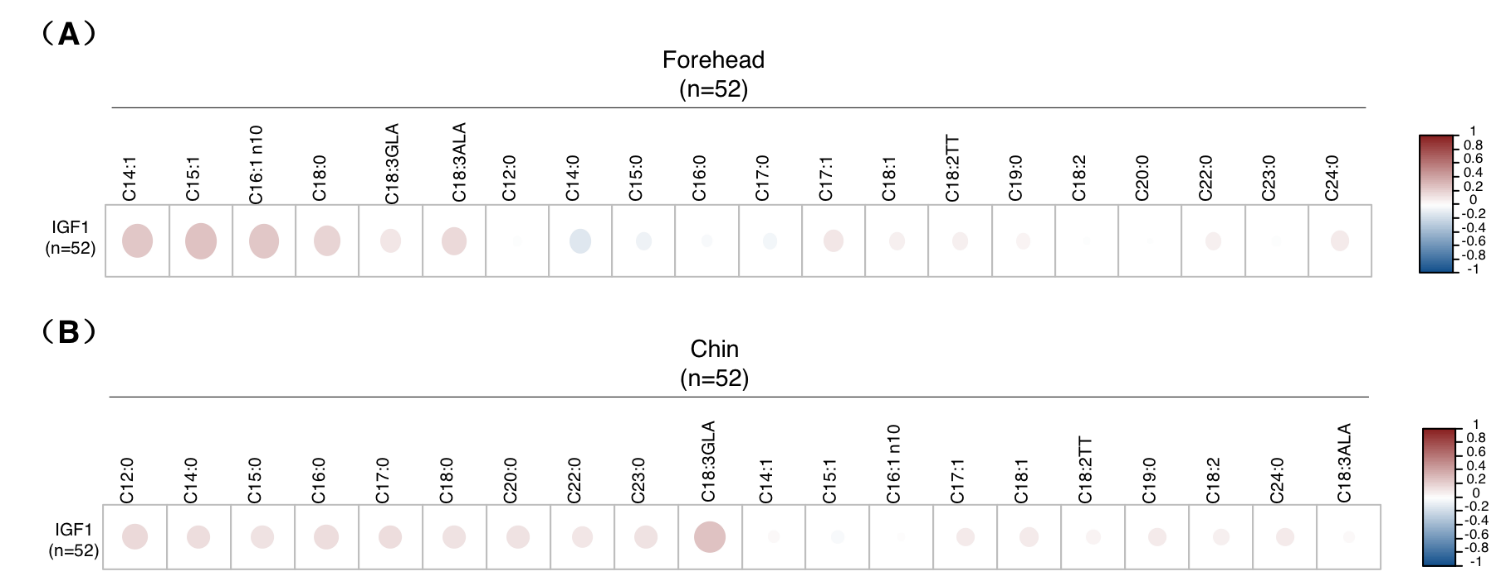


**Supplementary Figure 2.** **Correlations between IGF1 levels and FA of sebum from forehead (A) and chin (B) in female subjects.**
